# Transport Pathways and Kinetics of Cerebrospinal Fluid Tracers in Mouse Brain Observed by Dynamic Contrast-Enhanced MRI

**DOI:** 10.21203/rs.3.rs-2544475/v1

**Published:** 2023-02-07

**Authors:** Yuran Zhu, Guanhua Wang, Chaitanya Kolluru, Yuning Gu, Huiyun Gao, Jing Zhang, Yunmei Wang, David L. Wilson, Xiaofeng Zhu, Chris A. Flask, Xin Yu

**Affiliations:** Case Western Reserve University; University of Michigan; Case Western Reserve University; Case Western Reserve University; Case Western Reserve University; Case Western Reserve University; Case Western Reserve University; Case Western Reserve University; Case Western Reserve University; Case Western Reserve University; Case Western Reserve University

**Keywords:** Cerebrospinal fluid, interstitial fluid, glymphatic pathway, CSF transport, subarachnoid space

## Abstract

**Background:**

Recent studies have suggested the glymphatic system as a solute transport pathway and waste removal mechanism in the brain. Imaging intracisternally administered tracers provides the opportunity of assessing various aspects of the glymphatic function. Dynamic contrast-enhanced magnetic resonance imaging (DCE-MRI) allows the evaluation of both the kinetics and spatial distribution of tracer transport in the whole brain. However, assessing mouse glymphatic function by DCE-MRI has been challenged by the small size of a mouse brain and the limited volume of fluids that can be delivered intracisternally without significantly altering the intracranial pressure. Further, previous studies in rats suggest that assessment of glymphatic function by DCE-MRI is dependent on the molecular size of the contrast agents.

**Methods:**

We established and validated an intracisternal infusion protocol in mice that allowed the measurements of the entire time course of contrast agent transport for 2 hours. The transport kinetics and distribution of three MRI contrast agents with drastically different molecular weights (MWs): Gd-DTPA (MW=661.8 Da, n=7), GadoSpin-P (MW=200 kDa, n=6), and oxygen-17 enriched water (H_2_^17^O, MW=19 Da, n=7), were investigated.

**Results:**

The transport of H_2_^17^O was significantly faster and more extensive than the two gadolinium-based contrast agents. Time-lagged correlation analysis and clustering analysis comparing the kinetics of Gd-DTPA and H_2_^17^O transport also showed different cluster patterns and lag time between different regions of the brain, suggesting different transport pathways for H_2_^17^O because of its direct access to parenchymal tissues via the aquaporin-4 water channels. Further, there were also significant differences in the transport kinetics of the three tracers to the lateral ventricles, which reflects the differences in forces that drive tracer transport in the brain.

**Conclusions:**

Comparison of the transport kinetics and distribution of three MRI contrast agents with different molecular sizes showed drastically different transport profiles and clustering patterns, suggesting that the transport pathways and kinetics in the glymphatic system are size-dependent.

## Background

Recent evidence suggests that the exchange of the cerebrospinal fluid (CSF) with the parenchymal interstitial fluid (ISF) occurs via a highly regulated, brain-wide pathway [1]. The glymphatic model proposes that CSF in the subarachnoid space is driven by cerebral arterial pulsation along the perivascular space surrounding penetrating arteries [2], and its influx into the parenchyma is facilitated by the astroglial water channel aquaporin-4 (AQP4) located on the vascular endfeet [3]. The bulk flow from the influx of CSF into the parenchymal interstitium provides an efficient clearance route for metabolic by-products and other toxic wastes from the brain [4]. The transport of various CSF tracers has been studied extensively in rodent models to evaluate many pathophysiological factors that may impact CSF transport and CSF-ISF exchange, including the sleep-wake cycle, anesthesia, body postures, and cardiac function [5–10]. Further, impaired glymphatic function has been indicated in various disease conditions such as stroke, diabetes, traumatic brain injury, Alzheimer’s disease, and other dementias [11–16].

Glymphatic flow and its dependence on AQP4 were first characterized in vivo by two-photon microscopy using fluorescent tracers with different molecular weights (MWs) [1]. Subsequent studies on four different lines of AQP4 knockout mice further confirmed the critical role of AQP4 in solute transport in the glymphatic system [3]. Dynamic contrast-enhanced MRI (DCE-MRI) provides the opportunity to assess both the kinetics and spatial distribution of CSF tracers in the whole brain [17, 18]. Iliff and colleagues were the first to use DCE-MRI to evaluate the transport of MRI contrast agents in the glymphatic system in rat brains [19]. By comparing the transport of two gadolinium-based contrast agents (GBCAs) with different molecular sizes (Gd-DTPA, MW = 938 Da; GadoSpin, MW = 200 kDa), they showed that GadoSpin transport was confined to the subarachnoid space and CSF conduits, while Gd-DTPA was able to participate in CSF-ISF exchange. However, a limitation of using Gd-DTPA as a CSF tracer is that Gd-DTPA has limited penetration to the parenchyma because of its large molecular size, which may lead to an underestimation of the CSF-ISF exchange. Indeed, a recent study by Alshuhri et al compared the transport of Gd-DTPA and oxygen-17 (^17^O) enriched water (H_2_^17^O, MW = 19 Da) in rats and reported significantly faster transport kinetics of H_2_^17^O, which is exchanged into the parenchyma directly via AQP4 [20]. These studies suggest that the assessment of glymphatic function by DCE-MRI is dependent on the molecular size of the contrast agent, and using H_2_^17^O as a CSF tracer provides a unique opportunity to directly evaluate CSF-ISF exchange via AQP4.

Following these foundational DCE-MRI studies in rats, interest in evaluating the glymphatic function in mice is growing, largely due to the availability of genetically manipulated mouse models [3, 6, 11, 21–25]. Assessing mouse glymphatic function by DCE-MRI has been challenged by the small size of a mouse brain and the limited volume (< 20 μL) of fluids that can be delivered intracisternally without significantly altering the intracranial pressure (ICP) [15, 26]. Early studies on mice used the protocol of administering contrast agents on the bench to allow close monitoring of the infusion process and to visually ensure proper sealing of the infusion site. While subsequent MRI scanning enabled assessing tracer transport at a later stage, such an approach inevitably missed the initial phase of contrast agent transport. More recently, Stanton et al successfully performed mouse DCE-MRI studies with in-scanner delivery of GBCA [9]. By monitoring the dynamics of GBCA transport for an hour, their results show that contrast enhancement peaked within 20 min of infusion in most of the regions characterized, confirming the importance of delineating the kinetics of contrast agent transport at the early phase of infusion.

To build upon these prior studies, the goal of the current study was to evaluate the size-dependent transport kinetics and distribution of MRI contrast agents in the mouse glymphatic system. We first established and validated an intracisternal infusion protocol in mice that allowed the measurements of the entire time course of contrast agent transport for 2 hours. DCE-MRI studies were performed to compare the transport kinetics of Gd-DTPA, GadoSpin, and H_2_^17^O. Atlas-registered image analysis confirmed significantly faster transport of H_2_^17^O than Gd-DTPA, as well as drastically different transport profiles of the three tracers from cisterna magna to the lateral ventricles. Further, clustering analysis comparing the kinetic profiles of contrast agent induced signal changes showed a remarkable difference between H_2_^17^O and Gd-DTPA transport, suggesting different transport pathways for these two contrast agents.

## Materials And Methods

### Animals

The animal protocol was approved by the Institutional Animal Care and Use Committee of Case Western Reserve University. The experiments were performed on 13- to 15-week-old male C57BL/6J mice (Jackson Laboratories, Bar Harbor, ME, US). The average body weight at the time of MRI scan was 29.4 g. The animals were housed in a temperature- and humidity-controlled environment with ad libitum access to food and water and a 12-hour light-dark cycle.

### Cisterna magna cannulation

The animal was anesthetized with 3% isoflurane mixed with 100% O_2_ in an induction chamber and transferred to a stereotaxic frame with the head secured by ear bars and a tooth bar. Anesthesia was maintained with 1.5% isoflurane in 100% O_2_ delivered via a nose cone. The body temperature was maintained at ~ 37°C with a heating tape attached to the surface of the stereotaxis frame. A midline dorsal neck incision was made to expose the dura mater. A small durotomy was made using a 30-gauge needle to expose the cisterna magna. A polyethylene micro-catheter (0.13 mm ID × 0.25 mm OD, Scientific Commodities, Lake Havasu City, AZ, US) was inserted into the intrathecal space and secured with cyanoacrylate glue. Tracer delivery via cisterna magna was first confirmed with bright-field microscopy of Evans blue and cryoimaging of CF594 hydrazide and FITC-dextran (Fig. S1).

### MRI protocol

MRI studies were performed on a horizontal bore 9.4T preclinical scanner (Bruker Biospin, Billerica, MA, US) using a 35-mm volume coil. Mice were randomly assigned to three groups with intracisternal infusion of 1) 12.5 mM Gd-DTPA (n = 7; MW = 661.8 Da; Mallinckrodt, St Louis, MO, US); 2) 4.17 mM GadoSpin-P (n = 6; MW = 200 kDa; Miltenyi Biotec, Bergisch Gladbach, Germany); and 3) 90% enriched H_2_^17^O (n = 7; MW = 19 Da; NUKEM Isotopes, Alzenau, Germany). After the cannulation, the mouse was transferred to an MRI-compatible cradle and placed in a prone position. Anesthesia was maintained with 1 to 1.5% isoflurane delivered via a nose cone. Respiration rate and body temperature were monitored during the MRI scan. The respiration rate was maintained within 90 to 110 breaths per minute (bpm) by adjusting the anesthesia level. The body temperature was maintained at ~ 37°C by blowing warm air into the scanner through a feedback control system (SA Instruments, Stony Brook, NY, US). After the initial setup, an anatomic scan was acquired at baseline using a 3D spin-echo sequence with the following parameters: TR/TE, 50/8.1 ms; FOV, 20×16×14 mm^3^; matrix size, 100×80×70; NAV, 2. Total scan time was ~ 9 min. Subsequently, DCE-MRI acquisition was started, and 10 μL of contrast agent was infused at a rate of 1 μL/min (10 min total infusion time).

The transport of Gd-DTPA and GadoSpin was tracked dynamically using a T_1_-weighted 3D FLASH sequence with the following parameters: TR/TE, 50/2.5 ms; flip angle, 15°; FOV, 20×16×14 mm^3^; matrix size, 100×80×70; NAV, 1, leading to an isotropic resolution of 200 μm and a temporal resolution of 5 min. A single baseline scan and 25 dynamic scans were acquired before, during, and after contrast agent infusion for 130 min.

H_2_^17^O transport was imaged with a T_2_-weighted multi-slice RARE sequence. A total of 28 slices were acquired with TR, 2500 ms; FOV, 20×16 mm^2^; matrix size, 100×80; slice thickness, 0.5 mm. 8 echoes were acquired in each TR, leading to an effective TE of 31 ms. Acquisition time for a single-average dataset was 25 s. A total of 312 images were acquired continuously for 130 min with the first 12 images acquired as baseline.

To evaluate the significance of H_2_^17^O exchange across the blood-brain barrier (BBB), blood was collected from a subset of mice infused with H_2_^17^O at the end of DCE-MRI scan (n = 4). Additional experiments were performed on the bench to collect blood from mice 10 min after the completion of H_2_^17^O infusion (i.e., 20 min from the start of H_2_^17^O infusion, n = 3), as well as from mice without H_2_^17^O infusion (n = 3). The blood samples were centrifuged at 2400 RPM for 20 min and the plasma was extracted. The T_2_ values of the plasma samples were measured using a single-slice Car-Purcell-Meiboom-Gill (CPMG) sequence with the following acquisition parameters: TR, 10 s; FOV, 20×20 mm^2^; matrix size, 128×128; slice thickness, 1 mm. A total of 64 echoes were acquired with an evenly spaced echo time of 8 ms.

### MRI image analysis

All images and data analyses were performed using either in-house developed or open-source software in MATLAB (MathWorks, Natick, MA, US) or Python (Python Software Foundation, v.3.0). Single-average T_1_-weighted images were reconstructed to delineate the kinetics and distribution of Gd-DTPA and GadoSpin transport. For the analysis of H_2_^17^O transport, T_2_-weighted images were reconstructed using 6 averages, resulting in a temporal resolution of 2.5 min. Motion correction was performed by registering the dynamic images to the anatomic image acquired at baseline via affine transformation using the open-source toolkit, Advanced Normalization Tools [27–29].

Following motion correction, image segmentation was performed by co-registering images to an MRI mouse brain atlas [30]. Specifically, a representative animal was selected from each contrast agent group, and the anatomic images of the representative animal were registered to the atlas through affine and deformable transformation. Subsequently, images from the remaining animals in each contrast agent group were registered to that of the representative animal, followed by the generation of averaged dynamic images and time maximum intensity projection maps (tMIPs).

A total of 20 regions of interest (ROIs) were generated from the co-registered brain atlas, covering the intracisternal infusion and transport pathways, the brain parenchyma, and the lateral ventricles. Mean signal intensity in each ROI was calculated, followed by the subtraction of the baseline signal for the entire dynamic series. Subsequently, the maximal signal from a small region surrounding the infusion catheter in the cisterna magna was used as the “input function” to normalize the time course of signal changes in each ROI.

### Clustering analysis

A correlation-matrix-based hierarchical clustering (CMBHC) method was used to analyze the time courses of Gd-DTPA and H_2_^17^O transport among different ROIs [31]. Specifically, time-lagged cross-correlation analysis was performed to determine the similarity of the kinetics of contrast agent transport between two ROIs, as well as the lag time corresponding to the maximal cross-correlation coefficient (mCC) [32]. Subsequently, the “dissimilarity”, defined as 1-mCC, was used to quantify the distance between two ROIs. A dendrogram representing the hierarchical structure of the mCC matrix with complete linkage was generated using the open-source library SciPy [33]. Bootstrap analysis using the Pvclust package was performed to assess the uncertainty in clustering analysis [34]. A bootstrap replication of 1000 was used to calculate the approximately unbiased (AU) p-values and the bootstrap probability (BP) values. Visualizations of ROIs in each cluster were created using the Allen mouse brain atlas and brainrender [35, 36].

## Results

### Transport of contrast agents in the whole brain

**Figure 1:**
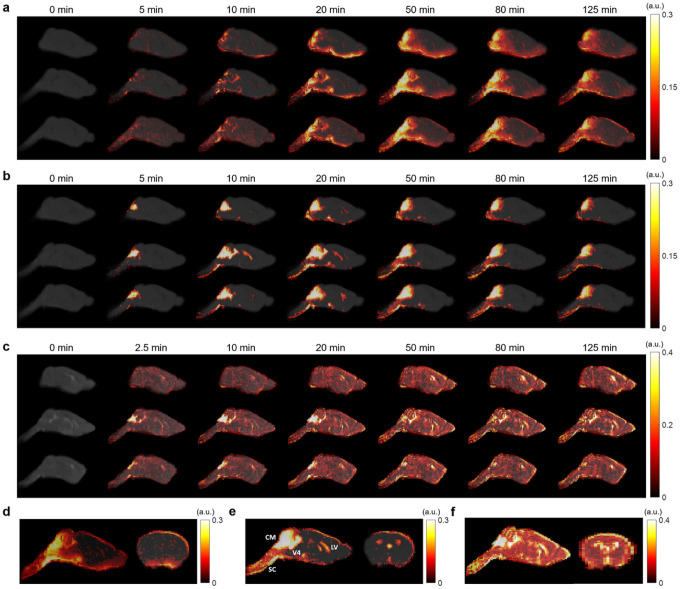
Contrast agent distribution. **a-c** Representative sagittal views of group-averaged images, overlaid with signal changes from the baseline at selected time points, of mice infused with Gd-DTPA (**a**), GadoSpin (**b**), and H_2_^17^O (**c**), respectively. **d-f** Time maximum intensity projection maps of representative sagittal and axial views of mice infused with Gd-DTPA (**d**), GadoSpin (**e**), and H_2_^17^O (**f**), respectively. CM, V4, LV, and SC in (**e**) indicate the contrast infusion site at cisterna magna, the fourth ventricle, the lateral ventricle, and the spinal cord, respectively.

Figure 1 shows representative sagittal slices of signal changes over the 2-hour DCE-MRI scans for each contrast agent. The signal changes showed clear differences in the transport dynamics of the three contrast agents (Figs. 1 a–c). The tMIPs further demonstrated the differences in contrast agent distributions during the entire time course of DCE-MRI scans (Figs. 1 d–f). Following the infusion of the contrast agents at cisterna magna, contrast enhancement in the cerebellum region proximal to the infusion site can be observed within 5 min of infusion (CM in Fig. 1 e). Subsequently, the transport of all three contrast agents to the fourth ventricle (V4), as well as along the subarachnoid space on the ventral surface of the brain, can be appreciated. Gd-DTPA was also transported into the parenchyma towards the dorsal direction after 15 min (Figs. 1a and 1d). In contrast, the transport of GadoSpin was primarily confined in the ventricles and the subarachnoid space (Figs. 1b and 1e). Compared to the two GBCAs, the transport of H_2_^17^O was more extensive and its distribution at the dorsal side of the brain was more pronounced (Figs. 3c and 3f). Finally, the downstream flow to the spinal cord (SC) was detectable for all three contrast agents.

### Dynamics of contrast agent transport

**Figure 2:**
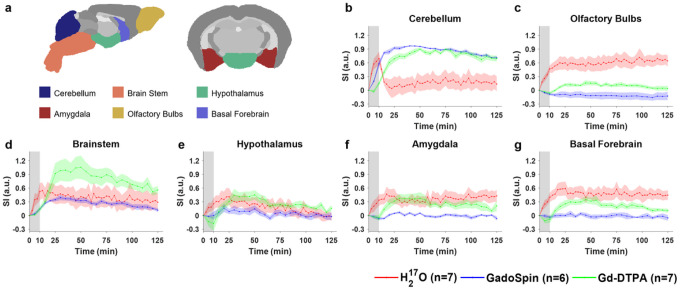
Dynamics of contrast agent transport in cerebellum and ventral brain regions. **a** Segmentation of selected ROIs. **b-g** Time courses of signal changes in the selected ROIs. Gray bands indicate the time period of contrast agent infusion. Red, blue, and green lines represent the mean time courses of signal changes induced by H_2_^17^O, GadoSpin, and Gd-DTPA, respectively. Shaded areas represent standard errors.

**Figure 3:**
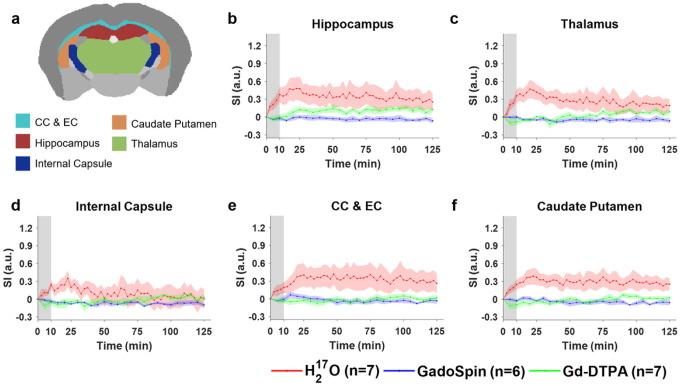
Dynamics of contrast agent transport in deep brain regions. **a** Segmentation of selected ROIs. **b-f** Time courses of signal changes in the selected ROIs. Gray bands indicate the time period of contrast agent infusion. CC & EC: Corpus callosum and external capsule. Red, blue, and green lines represent the mean time courses of signal changes induced by H_2_^17^O, GadoSpin, and Gd-DTPA, respectively. Shaded areas represent standard errors.

**Figure 4:**
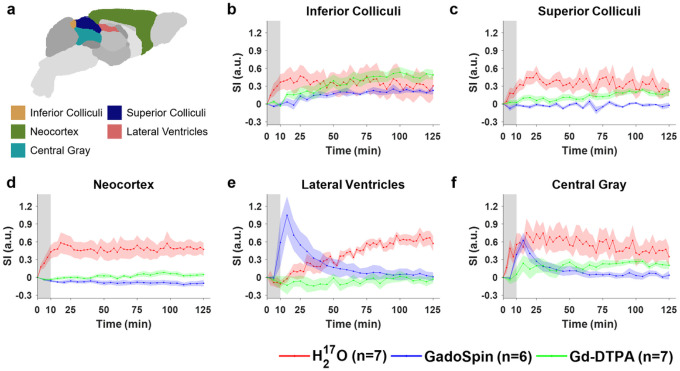
Dynamics of contrast agent transport in dorsal brain, lateral ventricles and central gray regions. **a** Segmentation of selected ROIs. **b-f** Time courses of signal changes in the selected ROIs. Gray bands indicate the time period of contrast agent infusion. Red, blue, and green lines represent the mean time courses of signal changes induced by H_2_^17^O, GadoSpin, and Gd-DTPA, respectively. Shaded areas represent standard errors.

Shown in Figs. 2 to 4 and Fig. S2 are the time courses of normalized signal changes in selected ROIs covering the cerebellum and the ventral brain surface (Fig. 2), the deep brain (Fig. 3 and Fig. S2), the dorsal brain and the ventricular regions (Fig. 4). The dynamics of signal changes of the three contrast agents differ drastically in both the magnitude and the rate of change over time. In cerebellum (Fig. 2b), an ROI proximal to the site of contrast agent infusion, all three contrast agents showed rapid uptake, with H_2_^17^O being the fastest. While the accumulation of the two GBCAs remained high, H_2_^17^O exhibited very rapid clearance in the cerebellum, suggesting fast transport of H_2_^17^O from the infusion site to the rest of the brain. The transport of H_2_^17^O was also faster than that of Gd-DTPA in all the ROIs. In the deep brain and dorsal regions (Figs. 3 and 4), the transport of Gd-DTPA was further delayed compared to that in the ventral brain regions, with significantly reduced magnitude of signal enhancement that did not reach steady-state over a time period of ~ 2 hrs, while H_2_^17^O transport in these regions was significantly faster and of a higher magnitude.

Compared to Gd-DTPA and H_2_^17^O, GadoSpin uptake only occurred in the ventricles and a few regions directly adjacent to the subarachnoid or large perivascular spaces, such as brainstem, inferior colliculi, and central gray. In the lateral ventricles (Fig. 4e), GadoSpin showed a prominent rapid uptake that peaked within 15 min after its infusion, followed immediately by a rapid clearance. In contrast, a delayed but progressive accumulation of H_2_^17^O was observed, while the transport of Gd-DTPA to the lateral ventricles was negligible.

### Clustering of ROIs

**Figure 5:**
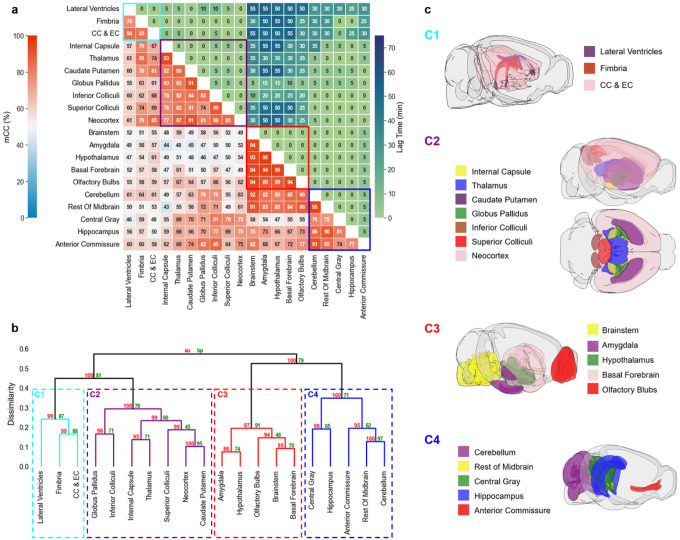
Correlation-matrix-based clustering analysis of Gd-DTPA transport. **a**Matrix of maximal cross-correlation (lower left) and lag time (upper right). **b** Cluster dendrogram with bootstrap analysis. Values at nodes are AU p-values (red, left) and BP values (green, right), respectively. Clusters with a dissimilarity value of <0.4 are indicated by the rectangles in (**a**) and (**b**). **c** Outlines of ROIs in each cluster. CC & EC: Corpus callosum and external capsule.

**Figure 6:**
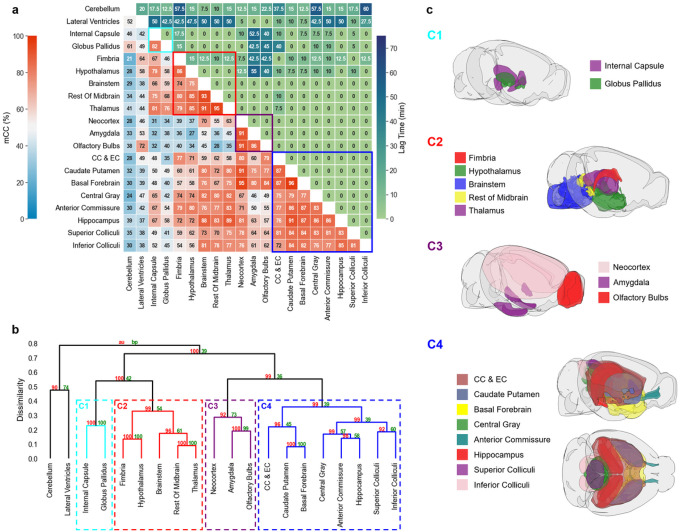
Correlation-matrix-based clustering analysis of H_2_^17^O transport. **a** Matrix of maximal cross-correlation (lower left) and lag time (upper right). **b** Cluster dendrogram with bootstrap analysis. Values at nodes are AU p-values (red, left) and BP values (green, right), respectively. Clusters with a dissimilarity value of <0.4 are indicated by the rectangles in (**a**) and (**b**). **c** Outlines of ROIs in each cluster. CC & EC: Corpus callosum and external capsule.

Results of correlation-matrix-based hierarchical clustering analysis of Gd-DTPA and H_2_^17^O transport are shown in Figs. 5 and 6, respectively. Figures 5a and 6a show the mCC and lag time between each pair of ROIs. A large mCC value between two ROIs suggests a high similarity in the dynamic profiles of signal enhancement while the lag time indicates a relative delay in contrast agent transport between the two ROIs. Dendrograms from clustering analysis are shown in Figs. 5b and 6b. Four clusters with a dissimilarity value of < 0.4 were identified for both Gd-DTPA and H_2_^17^O. Bootstrap analysis with a replication of 1000 showed an AU p-value of 0.92 to 1. These clusters are indicated by the rectangles in the correlation matrices in Figs. 5a and 6a, and their anatomical locations are outlined in Figs. 5c and 6c.

For Gd-DTPA, all five ROIs along the ventral brain surface were classified into the same cluster (C3 in Fig. 5c). The mCC values for this cluster were >93% for the four ROIs in the posterior to midbrain regions and decreased slightly to 89% in the olfactory bulbs in the anterior region (Fig. 5a, red rectangle). Further, the lag time was 0 among all these five ROIs, suggesting fast Gd-DTPA transport along the ventral surface of the brain. The remaining three clusters were grouped by their proximity to the subarachnoid space or ventral brain. While C4 was anatomically adjacent to these regions, C1 and C2 were located distally in the deep brain and dorsal regions. Correlation analysis of the ROIs in C1 and C2 with those in the ventral surface (C3) also showed the lowest mCC, as well as a longer lag time ranging from 25 to 55 min (Fig. 5a). In contrast, the lag time between ROIs in C3 and C4 was 0 except for anterior commissure (5 min).

H_2_^17^O transport showed distinctly different patterns in correlation matrix, lag time, and clustering from those of Gd-DTPA (Fig. 6). The five ROIs along the ventral surface were classified into three different clusters (C2–C4) instead of one. The clusters were primarily grouped by their proximity to the infusion site, with two clusters (C1 and C2) located in the posterior to midbrain regions and the other two clusters (C3 and C4) located in the midbrain to anterior regions. Lag time between ROIs in the same cluster was 0 except for the fimbria, the most anterior ROI in C2, which showed a lag time of 10–15 min from other ROIs in C2. Further, the lag time between two ROIs in different clusters was typically <15 min. Only a few ROIs, such as the cerebellum, lateral ventricles, amygdala, and olfactory bulbs, showed a longer lag time, which can be attributed to the differences in the kinetic profiles rather than a delay in H_2_^17^O transport, as indicated by the very low mCC values (^40%) between these ROIs.

### Impact of H_2_^17^O exchange across BBB

To evaluate the impact of the exchange of intracisternal H_2_^17^O with systemic circulation, blood samples were collected from mice at 20 min (n = 3) and 125 min (n = 4) after H_2_^17^O infusion, as well as from mice without H_2_^17^O infusion (n = 3). T_2_ measurements of the plasma samples did not show significant differences among these three groups (Fig. S3), suggesting that H_2_^17^O exchange across BBB did not significantly alter the T_2_ of circulating blood.

## Discussion

This study investigated the size-dependent transport of intracisternally administered CSF tracers in mouse brain using DCE-MRI. Consistent with previous observations in rats [19], Gd-DTPA showed relatively fast transport kinetics along the ventral surface of the brain but delayed and reduced transport into the deep brain and dorsal regions. GadoSpin transport showed limited penetration to all parenchymal regions of the brain. Compared to the two GBCAs, the transport of H_2_^17^O was both faster and more extensive, which is also in agreement with a previous study in rats [20]. These findings were further supported by time-lagged cross-correlation analysis in which Gd-DTPA showed a much longer lag time between ROIs in the ventral surface versus those in the deep brain and dorsal regions as compared to H_2_^17^O. In addition, clustering analysis also showed different patterns of transport between Gd-DTPA and H_2_^17^O. Further, the three tracers also showed drastically different transport kinetics from cisterna magna to the lateral ventricles.

Previous studies of rat glymphatic function have employed pixel-based clustering analysis to identify regions with similar contrast enhancement features [19, 37]. Using the area-under-the-curve in k-means clustering algorithm, Iliff et al demonstrated deeper penetration of Gd-DTPA to parenchymal tissues than GadoSpin [19]. In an effort to model the glymphatic system using local input function, Davoodi-Bojd et al used the derivatives of the time signal curve to identify clusters with similar profiles of contrast enhancement [37]. In the current study, we performed ROI-based hierarchical clustering analysis using mCC-derived parameters as a measure of “distance” between two ROIs or clusters. Such an approach allowed the identification of ROIs with similar kinetic profiles over the entire time course (2 hours). While lag time was not used as a parameter in clustering analysis, most of the ROIs within a cluster showed minimal lag time for both Gd-DTPA and H_2_^17^O, suggesting that this approach is robust in identifying ROIs along the transport pathway.

The clustering of Gd-DTPA transport was highly consistent with that by Iliff et al using a pixel-based clustering approach. In particular, all five ROIs along the ventral surface were identified as a single cluster while the remaining ROIs in the parenchymal tissues were clustered by their proximity to the subarachnoid space and ventral surface of the brain. More importantly, ROIs in these clusters also showed a prolonged lag time from the ROIs in the ventral cluster. These analyses suggest that Gd-DTPA transport occurred initially via the perivascular network, followed by its penetration into the parenchyma. In contrast, H_2_^17^O transport showed dramatically different clustering patterns compared to Gd-DTPA, with the five ventral ROIs distributed in three different clusters. In general, clustering of H_2_^17^O transport followed a pattern of posterior to anterior while Gd-DTPA was along the ventral surface towards the dorsal direction. These different clustering patterns reflect different transport pathways for H_2_^17^O because of its direct access to parenchymal tissues via AQP4.

A potential confounding factor in H_2_^17^O transport is the exchange of water across the BBB. Since systemic circulation is much faster than CSF flow [38, 39], its participation in H_2_^17^O transport may significantly accelerate the distribution of H_2_^17^O to the whole brain. To evaluate the impact of H_2_^17^O exchange across BBB, we measured the T_2_ of plasma collected at two time points, one at the time when H_2_^17^O-induced T_2_ change reached peak in parenchyma (20 min) and one at the end of the scan protocol (125 min). Our measurements showed no significant difference in plasma T_2_ at both time points compared to samples collected from mice without H_2_^17^O infusion. This lack of change in plasma T_2_ suggests that the intracisternal administration of a small volume of H_2_^17^O (10 μL) had a negligible effect on plasma H_2_^17^O concentration. As such, it is unlikely that systemic circulation played a significant role in the observed fast H_2_^17^O transport in the current study. However, further investigations are warranted to evaluate whether water exchange across BBB can serve as an “efflux route” for H_2_^17^O and whether the influx of unlabeled water from systemic circulation can “dilute” the effect of H_2_^17^O-induced T_2_ change in the parenchyma, both may lead to an underestimation of H_2_^17^O transport if not accounted for.

A recent study using confocal microscopy and fluorescein-labeled dextran (MW = 500 kDa) as a CSF tracer has suggested that tracers administered via cisterna magna can be transported to the lateral ventricles via the interpeduncular cistern [40]. However, such observation was not unequivocally supported by a DCE-MRI study using Gd-DOTA (MW = 661.8 Da) as the tracer, in which only one rat with an abnormally enlarged third ventricle exhibited Gd-DOTA uptake in the lateral ventricles [41]. In our current study, mice also showed negligible uptake of Gd-DTPA into the lateral ventricles. However, there was a rapid uptake of GadoSpin into the lateral ventricles that peaked at the end of its infusion, and it was immediately followed by a rapid clearance. Interestingly, despite its fast transport to the parenchymal tissues, H_2_^17^O showed a slow but progressive accumulation of H_2_^17^O in the lateral ventricles (Fig. 4e). Considering that cisterna magna is at the downstream of CSF circulation from the fourth ventricle [42], direct transport of CSF tracers from cisterna magna to the lateral ventricles would require a reversal of the pressure gradient. The transport of GadoSpin into the lateral ventricles suggests that intracisternal infusion may have caused such a transient reversal of the pressure gradient between the cisterna magna and the lateral ventricles, and the rapid clearance of GadoSpine appears to be consistent with the restoration of the normal pressure gradient immediately after the infusion. On the other hand, the delayed transport of H_2_^17^O and the negligible transport of Gd-DTPA into the lateral ventricles suggest that the pressure gradient and the transport impedance for these two contrast agents may have favored their transport from the subarachnoid space into the parenchymal tissues. As such, the delayed H_2_^17^O uptake into the lateral ventricles may have occurred at the parenchymal-ventricular interface following its transport to the parenchyma. However, due to the slow and reduced penetration of Gd-DTPA into the parenchyma, Gd-DTPA did not reach the parenchymal-ventricular interface in a significant amount, leading to its negligible uptake into the lateral ventricles. Further investigations rooted in the fluid dynamics of CSF circulation are warranted to gain more quantitative insight of these observations.

There are several limitations in the current study. First, the use of T_1_- or T_2_-weighted images in DCE-MRI is a semiquantitative approach such that signal changes from the baseline can only serve as a proxy of contrast agent concentration. While the concentrations of gadolinium ions (Gd^2+^) were matched for the two GBCAs, a direct comparison of contrast agent concentrations is not possible because of the nonlinear relationship between signal changes and contrast agent concentrations. As such, the current study focused on evaluating the kinetics of contrast agent induced signal changes. To account for inter-subject variations, we normalized the signal changes by using the maximal signal surrounding the infusion site as the common denominator. While this approach has enabled the comparison of relative signal changes in different ROIs, flux quantification is challenged by the nonlinear relationship between signal change and contrast concentration. Second, due to hardware limitations, a lower spatial resolution was used in the current study to achieve adequate temporal resolution, which has led to the pronounced partial volume effect in a few ROIs. For example, the large signal increase in the central gray from mice infused with GadoSpin (Fig. 4e) is likely caused by GadoSpin uptake in the fourth ventricle. Improving the spatial resolution will reduce the partial volume effect and enable the segmentation of smaller ROIs in future studies. Finally, previous studies have shown that general anesthesia with isoflurane alone can impair the circulation of CSF through the brain while low-dose isoflurane with supplementary dexmedetomidine or ketamine/xylazine can enhance glymphatic transport [5, 6, 9]. Hence, an anesthetic regime that enhances glymphatic function can be more desirable in studying solute transport in the brain, especially when using tracers with a large molecular size such as Gd-DTPA.

## Conclusions

In summary, the current study established an intracisternal infusion protocol that enabled DCE-MRI measurements of the entire time course of contrast agent transport in the mouse glymphatic system. Comparison of the transport kinetics and distribution of three MRI contrast agents with different molecular sizes showed drastically different transport profiles and clustering patterns, suggesting that the transport pathways and kinetics in the glymphatic system are size-dependent.

## Data Availability

Experimental data, images, and code from this study are available upon request to the corresponding author.

## Abbreviations

CSF: cerebrospinal fluid
ISF: interstitial fluid
AQP4: aquaporin-4 water channel
ICP: intracranial pressure
BBB: blood-brain barrier
MW: molecular weights
DCE-MRI: dynamic contrast-enhanced magnetic resonance imaging
GBCA: gadolinium-based contrast agents
Gd-DTPA: gadopentetic acid
H_2_^17^O: oxygen-17 enriched water
CPMG: Car-Purcell-Meiboom-Gill
CMBHC: correlation-matrix-based hierarchical clustering
mCC: maximal cross-correlation coefficient
AU: approximately unbiased
BP: bootstrap probability
CM: cisterna magna
V4: fourth ventricle
SC: spinal cord

## References

[1] Iliff JJ, Wang M, Liao Y, Plogg BA, Peng W, Gundersen GA A Paravascular Pathway Facilitates CSF Flow Through the Brain Parenchyma and the Clearance of Interstitial Solutes, Including Amyloid β. Sci Transl Med [Internet]. 2012 [cited 2022 Jun 6];4. Available from: https://www.science.org/doi/10.1126/scitranslmed.3003748 10.1126/scitranslmed.3003748 PMC3551275 22896675

[2] Iliff JJ, Wang M, Zeppenfeld DM, Venkataraman A, Plog BA, Liao Y, Cerebral Arterial Pulsation Drives Paravascular CSF-Interstitial Fluid Exchange in the Murine Brain. J Neurosci. 2013;33:18190–9. 24227727 10.1523/JNEUROSCI.1592-13.2013 PMC3866416

[3] Mestre H, Hablitz LM, Xavier AL, Feng W, Zou W, Pu T, Aquaporin-4-dependent glymphatic solute transport in the rodent brain. eLife. 2018;7:e40070. 30561329 10.7554/eLife.40070 PMC6307855

[4] Nedergaard M. Garbage Truck of the Brain. Science. 2013;340:1529–30. 23812703 10.1126/science.1240514 PMC3749839

[5] Benveniste H, Lee H, Ding F, Sun Q, Al-Bizri E, Makaryus R, Anesthesia with Dexmedetomidine and Low-dose Isoflurane Increases Solute Transport via the Glymphatic Pathway in Rat Brain When Compared with High-dose Isoflurane. Anesthesiology. 2017;127:976–88. 28938276 10.1097/ALN.0000000000001888 PMC5685871

[6] Gakuba C, Gaberel T, Goursaud S, Bourges J, Di Palma C, Quenault A, General Anesthesia Inhibits the Activity of the “Glymphatic System. Theranostics. 2018;8:710–22. 29344300 10.7150/thno.19154 PMC5771087

[7] Hablitz LM, Vinitsky HS, Sun Q, Stager FF, Sigurdsson B, Mortensen KN, Increased glymphatic influx is correlated with high EEG delta power and low heart rate in mice under anesthesia. Sci Adv. 2019;5:eaav5447. 10.1126/sciadv.aav5447 PMC6392807 30820460

[8] Lee H, Xie L, Yu M, Kang H, Feng T, Deane R, The Effect of Body Posture on Brain Glymphatic Transport. J Neurosci. 2015;35:11034–44. 26245965 10.1523/JNEUROSCI.1625-15.2015 PMC4524974

[9] Stanton EH, Persson NDÃ, Gomolka RS, Lilius T, Sigurosson B, Lee H, Mapping of CSF transport using high spatiotemporal resolution dynamic contrast-enhanced MRI in mice: Effect of anesthesia. Magn Reson Med. 2021;85:3326–42. 33426699 10.1002/mrm.28645

[10] Xie L, Kang H, Xu Q, Chen MJ, Liao Y, Thiyagarajan M, Sleep Drives Metabolite Clearance from the Adult Brain. Science. 2013;342:373–7. 24136970 10.1126/science.1241224 PMC3880190

[11] Gaberel T, Gakuba C, Goulay R, De Lizarrondo SM, Hanouz J-L, Emery E, Impaired Glymphatic Perfusion After Strokes Revealed by Contrast-Enhanced MRI: A New Target for Fibrinolysis? Stroke. 2014;45:3092–6. 25190438 10.1161/STROKEAHA.114.006617

[12] Goulay R, Flament J, Gauberti M, Naveau M, Pasquet N, Gakuba C, Subarachnoid Hemorrhage Severely Impairs Brain Parenchymal Cerebrospinal Fluid Circulation in Nonhuman Primate. Stroke. 2017;48:2301–5. 28526764 10.1161/STROKEAHA.117.017014

[13] Iliff JJ, Chen MJ, Plog BA, Zeppenfeld DM, Soltero M, Yang L, Impairment of Glymphatic Pathway Function Promotes Tau Pathology after Traumatic Brain Injury. J Neurosci. 2014;34:16180–93. 25471560 10.1523/JNEUROSCI.3020-14.2014 PMC4252540

[14] Jiang Q, Zhang L, Ding G, Davoodi-Bojd E, Li Q, Li L, Impairment of the glymphatic system after diabetes. J Cereb Blood Flow Metab. 2017;37:1326–37. 27306755 10.1177/0271678X16654702 PMC5453454

[15] Kress BT, Iliff JJ, Xia M, Wang M, Wei HS, Zeppenfeld D, Impairment of paravascular clearance pathways in the aging brain: Paravascular Clearance. Ann Neurol. 2014;76:845–61. 25204284 10.1002/ana.24271 PMC4245362

[16] Rasmussen MK, Mestre H, Nedergaard M. The glymphatic pathway in neurological disorders. Lancet Neurol. 2018;17:1016–24. 30353860 10.1016/S1474-4422(18)30318-1 PMC6261373

[17] Jiang Q. MRI and glymphatic system. Stroke Vasc Neurol. 2019;4:75–7. 31338214 10.1136/svn-2018-000197 PMC6613867

[18] Taoka T, Naganawa S. Glymphatic imaging using MRI. J Magn Reson Imaging. 2020;51:11–24. 31423710 10.1002/jmri.26892

[19] Iliff JJ, Lee H, Yu M, Feng T, Logan J, Nedergaard M, Brain-wide pathway for waste clearance captured by contrast-enhanced MRI. J Clin Invest. 2013;123:1299–309. 23434588 10.1172/JCI67677 PMC3582150

[20] Alshuhri MS, Gallagher L, Work LM, Holmes WM. Direct imaging of glymphatic transport using H217O MRI. JCI Insight. 2021;6:e141159. 10.1172/jci.insight.141159 PMC8262348 33857020

[21] Ma Q, Ries M, Decker Y, Müller A, Riner C, Bücker A, Rapid lymphatic efflux limits cerebrospinal fluid flow to the brain. Acta Neuropathol (Berl). 2019;137:151–65. 30306266 10.1007/s00401-018-1916-x PMC6338719

[22] Xue Y, Gursky Z, Monte B, Koundal S, Liu X, Lee H, Sustained glymphatic transport and impaired drainage to the nasal cavity observed in multiciliated cell ciliopathies with hydrocephalus. Fluids Barriers CNS. 2022;19:20. 35248089 10.1186/s12987-022-00319-x PMC8898469

[23] Xue Y, Liu X, Koundal S, Constantinou S, Dai F, Santambrogio L, In vivo T1 mapping for quantifying glymphatic system transport and cervical lymph node drainage. Sci Rep. 2020;10:14592. 32884041 10.1038/s41598-020-71582-x PMC7471332

[24] Zamani A, Walker AK, Rollo B, Ayers KL, Farah R, O’Brien TJ, Impaired glymphatic function in the early stages of disease in a TDP-43 mouse model of amyotrophic lateral sclerosis. Transl Neurodegener. 2022;11:17. 35287738 10.1186/s40035-022-00291-4 PMC8922788

[25] Zhang J, Zhao H, Xue Y, Liu Y, Fan G, Wang H, Impaired Glymphatic Transport Kinetics Following Induced Acute Ischemic Brain Edema in a Mouse pMCAO Model. Front Neurol. 2022;13:860255. 10.3389/fneur.2022.860255 PMC8970176 35370910

[26] Benveniste H, Lee H, Ozturk B, Chen X, Koundal S, Vaska P Glymphatic Cerebrospinal Fluid and Solute Transport Quantified by MRI and PET Imaging. Neuroscience. 2021;474:63–79. 33248153 10.1016/j.neuroscience.2020.11.014 PMC8149482

[27] Avants BB, Tustison NJ, Song G, Cook PA, Klein A, Gee JC. A reproducible evaluation of ANTs similarity metric performance in brain image registration. NeuroImage. 2011;54:2033–44. 20851191 10.1016/j.neuroimage.2010.09.025 PMC3065962

[28] Hübner NS, Mechling AE, Lee H-L, Reisert M, Bienert T, Hennig J, The connectomics of brain demyelination: Functional and structural patterns in the cuprizone mouse model. NeuroImage. 2017;146:1–18. 27845252 10.1016/j.neuroimage.2016.11.008

[29] Koch S, Mueller S, Foddis M, Bienert T, von Elverfeldt D, Knab F, Atlas registration for edema-corrected MRI lesion volume in mouse stroke models. J Cereb Blood Flow Metab. 2019;39:313–23. 28829217 10.1177/0271678X17726635 PMC6360485

[30] Ma Y, Hof PR, Grant SC, Blackband SJ, Bennett R, Slatest L, A three-dimensional digital atlas database of the adult C57BL/6J mouse brain by magnetic resonance microscopy. Neuroscience. 2005;135:1203–15. 16165303 10.1016/j.neuroscience.2005.07.014

[31] Liu X, Zhu X-H, Qiu P Chen W. A correlation-matrix-based hierarchical clustering method for functional connectivity analysis. J Neurosci Methods. 2012;211:94–102. 22939920 10.1016/j.jneumeth.2012.08.016 PMC3477851

[32] Siegel JS, Snyder AZ, Ramsey L, Shulman GL, Corbetta M. The effects of hemodynamic lag on functional connectivity and behavior after stroke. J Cereb Blood Flow Metab. 2016;36:2162–76. 26661223 10.1177/0271678X15614846 PMC5363662

[33] Virtanen P Gommers R, Oliphant TE, Haberland M, Reddy T, Cournapeau D, SciPy 1.0: fundamental algorithms for scientific computing in Python. Nat Methods. 2020;17:261–72. 32015543 10.1038/s41592-019-0686-2 PMC7056644

[34] Suzuki R, Shimodaira H. Pvclust: an R package for assessing the uncertainty in hierarchical clustering. Bioinformatics. 2006;22:1540–2. 16595560 10.1093/bioinformatics/btl117

[35] Lein ES, Hawrylycz MJ, Ao N, Ayres M, Bensinger A, Bernard A, Genome-wide atlas of gene expression in the adult mouse brain. Nature. 2007;445:168–76. 17151600 10.1038/nature05453

[36] Claudi F, Tyson AL, Petrucco L, Margrie TW, Portugues R, Branco T. Visualizing anatomically registered data with brainrender. eLife. 2021;10:e65751. 33739286 10.7554/eLife.65751 PMC8079143

[37] Davoodi-Bojd E, Ding G, Zhang L, Li Q, Li L, Chopp M, Modeling glymphatic system of the brain using MRI. NeuroImage. 2019;188:616–27. 30578928 10.1016/j.neuroimage.2018.12.039 PMC6401298

[38] Li J, Pei M, Bo B, Zhao X, Cang J, Fang F, Whole-brain mapping of mouse CSF flow via HEAP-METRIC phase-contrast MRI. Magn Reson Med. 2022;87:2851–61. 35107833 10.1002/mrm.29179 PMC9305925

[39] Pardridge WM. Drug transport in brain via the cerebrospinal fluid. Fluids Barriers CNS. 2011;8:7. 21349155 10.1186/2045-8118-8-7 PMC3042981

[40] van der Bedussi B, de Vos J, van Veen H, Siebes M, VanBavel E, Paravascular channels, cisterns, and the subarachnoid space in the rat brain: A single compartment with preferential pathways. J Cereb Blood Flow Metab. 2017;37:1374–85. 27306753 10.1177/0271678X16655550 PMC5453458

[41] Lee H, Mortensen K, Sanggaard S, Koch P Brunner H, Quistorff B, Quantitative Gd-DOTA uptake from cerebrospinal fluid into rat brain using 3D VFA-SPGR at 9.4T: Quantitative Gd-DOTA Uptake from CSF into Rat Brain. Magn Reson Med. 2018;79:1568–78. 28627037 10.1002/mrm.26779 PMC5736474

[42] Simon MJ, Iliff JJ. Regulation of cerebrospinal fluid (CSF) flow in neurodegenerative, neurovascular and neuroinflammatory disease. Biochim Biophys Acta BBA - Mol Basis Dis. 2016;1862:442–51. 10.1016/j.bbadis.2015.10.014 PMC4755861 26499397

